# Cellular uptake of magnetic nanoparticles imaged and quantified by magnetic particle imaging

**DOI:** 10.1038/s41598-020-58853-3

**Published:** 2020-02-05

**Authors:** Hendrik Paysen, Norbert Loewa, Anke Stach, James Wells, Olaf Kosch, Shailey Twamley, Marcus R. Makowski, Tobias Schaeffter, Antje Ludwig, Frank Wiekhorst

**Affiliations:** 1 Physikalisch-Technische Bundesanstalt, Berlin, Germany; 2 Charité - Universitätsmedizin Berlin, corporate member of Freie Universität Berlin, Humboldt-Universität zu Berlin, Berlin Institute of Health, Medizinische Klinik für Kardiologie und Angiologie, Campus Mitte, Berlin, Germany; 3 DZHK (German Centre for Cardiovascular Research), partner site Berlin, Berlin, Germany; 4 Technical University Munich, Munich, Germany; 5 Charité - Universitätsmedizin Berlin, corporate member of Freie Universität Berlin, Humboldt-Universität zu Berlin, Berlin Institute of Health, Klinik für Radiologie, Berlin, Germany

**Keywords:** Imaging techniques and agents, Magnetic properties and materials, Nanoparticles, Nanoparticles, Nanoscale biophysics, Kinetics, Cellular imaging, Extracellular matrix

## Abstract

Magnetic particle imaging (MPI) is a non-invasive, non-ionizing imaging technique for the visualization and quantification of magnetic nanoparticles (MNPs). The technique is especially suitable for cell imaging as it offers zero background contribution from the surrounding tissue, high sensitivity, and good spatial and temporal resolutions. Previous studies have demonstrated that the dynamic magnetic behaviour of MNPs changes during cellular binding and internalization. In this study, we demonstrate how this information is encoded in the MPI imaging signal. Through MPI imaging we are able to discriminate between free and cell-bound MNPs in reconstructed images. This technique was used to image and quantify the changes that occur *in-vitro* when free MNPs come into contact with cells and undergo cellular-uptake over time. The quantitative MPI results were verified by colorimetric measurements of the iron content. The results showed a mean relative difference between the MPI results and the reference method of 23.8% for the quantification of cell-bound MNPs. With this technique, the uptake of MNPs in cells can be imaged and quantified directly from the first MNP cell contact, providing information on the dynamics of cellular uptake.

## Introduction

Magnetic particle imaging (MPI) is a non-invasive technique capable of determining the spatial distribution of magnetic nanoparticles (MNPs) both *in-vivo* and *in-vitro*^1^. MPI achieves imaging and quantification detecting the non-linear dynamic magnetic response of MNPs exposed to multiple superimposed static and dynamic magnetic fields with a submillimetre spatial resolution. No background signals are generated by bone or tissue. The technique uses non-ionizing radiation and non-toxic nanoparticles to prevent tissue damage. MPI shows great potential for different biomedical applications, such as angiography, stem cell tracking, diagnosis of inflammatory diseases and cancer^2–11^. In inflammation-associated diseases, including cancer, MNPs accumulate preferentially in diseased tissue as a result of leaky vasculature thereby enabling imaging with MRI and MPI^12–16^. In diseased tissue, MNPs accumulate mainly in macrophages^17,18^. These phagocytic cells are a hallmark of tissue inflammation and their quantity is considered a marker of the severity of the disease^19–21^.

The magnetic response generated by MNPs is severely influenced by their local environment. In this respect, the changing magnetic properties of MNPs induced by their interaction with cells are a relevant factor for MPI. Previous studies using magnetic particle spectroscopy (MPS), have described and quantified the changes that occur in the dynamic magnetization of certain MNP systems upon interaction with living cells^22–26^. These effects may be caused by a variety of factors including the aggregation of particles in the surrounding solution, within the extra-cellular matrix or within various intracellular compartments. “Size-filtering” during cellular uptake and an increase in dipole-dipole interactions caused by a lower separation and mobility of the MNPs may also be influencing this phenomenon^25–28^. Typically, these signal changes lead to a deterioration of MPI images, resulting in lower sensitivity, decreased spatial resolution and more imaging artifacts^26,28–30^. However, if the unique magnetic signal patterns induced by the interaction of MNPs with cells are known, they can be incorporated into the image reconstruction to negate or minimize these negative effects. Additionally, this enables the possibility to search and separate these unique signal patterns generated by MNPs in specific environmental conditions. Such a technique, known as multi-color or multi-contrast MPI, has been previously demonstrated by separating the signals of different MNP systems under varying parameters such as temperature or viscosity^30–32^.

The aim of this work was to test the potential of imaging the internalization of MNPs into living cells with MPI. It was hypothesized, that based on different magnetic signals, the particle distributions of free vs cell-bound MNPs can be separated using MPI image reconstruction. Furthermore, due to the high temporal resolution of MPI, the processes involved in the cellular uptake of MNPs were imaged and quantified.

## Materials and Methods

### MPI acquisition

All MPI measurements were performed on a commercial, preclinical MPI scanner (Bruker MPI 25/50 FF) based on the field-free point (FFP) approach. The FFP is generated by a static magnetic gradient field with gradient strengths 0.6/0.6/1.2 T/m (*x*/*y*/*z*-direction). By superimposing oscillating magnetic drive fields with field amplitudes of 12/12/12 mT and frequencies 24.5/26.0/25.3 kHz the FFP is moved on a Lissajous trajectory through the field of view (FOV) of size 40/40/20 mm. Based on these magnetic field settings and the nonlinear dynamic magnetic properties of MNPs, higher harmonics of the excitation frequencies are generated and measured by a gradiometric receive coil^33^. The spectral patterns of the MPI signal (***u*)** depend on the MNP system, the environmental conditions and the spatial distribution of the MNPs within the FOV. A 3D image reconstruction of the MNP distribution (***c***) is performed by solving the following least-squares problem using the Kaczmarz algorithm with Tikhonov regularization (regularization parameter *λ*):

$${\Vert {\boldsymbol{S}}{\boldsymbol{c}}-{\boldsymbol{u}}\Vert }^{2}+\lambda {\Vert {\boldsymbol{c}}\Vert }^{2}\to min.$$

The SF (***S*)** contains information on how the environmental conditions and spatial position affect the measured MPI signal ***u***. The SFs used in this study were acquired experimentally by measuring small reference samples of MNPs with fixed environmental conditions at multiple positions all over the scanner’s FOV. For this approach to be accurate, it is necessary that all environmental conditions influencing the MPI signal during a measurement are the same as the conditions during the SF acquisition. If the environmental conditions of the MNPs change, it must be considered in the image reconstruction process. This can be achieved by acquiring multiple SFs (***S***_**1**_ and ***S***_**2**_) of MNPs at different environmental conditions. Thus, a combined SF can be used to reconstruct multiple particle distributions (***c***_**1**_ and ***c***_**2**_) of MNPs at given environmental conditions by adapting the least squares problem:

$${\Vert [{{\boldsymbol{S}}}_{1}{{\boldsymbol{S}}}_{2}][\begin{array}{c}{{\bf{c}}}_{1}\\ {{\bf{c}}}_{2}\end{array}]-{\bf{u}}\Vert }^{2}+{\rm{\lambda }}\Vert [\begin{array}{c}{{\bf{c}}}_{1}\\ {{\bf{c}}}_{2}\end{array}]\Vert \to \,{\rm{\min }}.$$

Assuming that changes in the dynamic magnetization induced by interactions of cells and MNPs can be described with a simple two-state system, two SFs were acquired under same field settings with a fluid sample (8 μL Synomag, iron oxide MNP, product nr. 103-02-301, LOT: 05218103-03 iron concentration c(Fe) = 2.75 mg/ml, micromod Nanopartikeltechnologie GmbH Rostock) diluted in phosphate-buffered saline (PBS)) and a cell sample (2 · 10^6^ THP-1 cells incubated with Synomag), respectively. This enables the reconstruction of two particle distributions representing the free and cell-bound MNP distributions. The term “cell-bound” MNPs includes each MNP that is either adsorbed to the cell surface or internalized by the cell. The choice of reconstruction parameters heavily influences the quantitative and qualitative MPI images. Therefore, we varied the reconstruction parameters over a certain range, taking a priori knowledge of the particle distributions into account to determine an optimal parameter set. A detailed description of this procedure, and a list of all measurement and reconstruction parameters used in this study can be found in the supplement.

### Cell cultivation and sample preparation

THP-1 cells (human acute monocytic leukemia cell line) obtained from the ATCC (Wesel, Germany) were cultured in suspension in a humidified incubator at 37 °C with a 5% CO_2_ concentration in RPMI medium 1640 (Invitrogen, Karlsruhe, Germany). Culture medium was supplemented with 10% fetal calf serum (FCS, Biochrom, Berlin, Germany), 100 U/ml penicillin, 100 µg/ml streptomycin (Invitrogen), and 2 mM L-glutamine (Invitrogen). Exact cell numbers were determined with a hemocytometer. Light microscopy: after treatment with iron oxide nanoparticles, cells were washed and spotted on glass slides and iron was detected by Prussian blue staining (2% potassium ferrocyanide in 1% HCl) followed by counterstain with Nuclear Fast Red. Two samples were analysed; one sample incubated without MNPs and one incubated with Synomag (c(Fe) = 0.5 mM) over a time of 15 min.

The cell sample for system function acquisition was prepared as follows: 2 · 10^6^ THP-1 cells suspended in 2 mL of RPMI containing 1% FCS were treated for 3 hours with 0.5 mM Synomag. After incubation cells were centrifuged for 3 min at 200 g and pellets were washed three times with 1 mL PBS to remove all unbound MNPs. After washing, resuspended cells were filled into a cubic 2 mm³ container (Bruker Biospin, Germany). Centrifugation for 3 min at 200 g led to the sedimentation of the cells in the lower chamber of the container. After SF acquisition the iron content of the pellet was quantified using the 1,10-phenanthroline-based iron assay as previously described^15^.

For the colorimetric quantification of Synomag uptake similar conditions as for the quantification with MPI were used. As described in the experimental setup: The indicated numbers of THP-1 cells were suspended in 100 µL PBS, and added in a 1.5 mL Eppendorf tube to 40 µL of Synomag diluted in PBS with an iron concentration of 2.75 mg/mL. After incubation the samples including cells were centrifuged for 3 min at 200 g. Supernatants were collected and evaporated to dryness using a Speedvac (Savant Instruments, NY, USA). Pellets were washed twice with 1 mL PBS and centrifuged for 3 min at 200 g. Iron content in the sediment of the supernatants and of the pellets was quantified using the 1, 10-phenanthroline-based iron assay.

### Experimental setup: *in-vitro* imaging

A vessel containing 40 µL of Synomag diluted in PBS was prepared with an iron concentration of 2.75 mg/mL and mounted to a sample holder, which can be moved to the centre of the MPI FOV. A set of THP-1 cells with varying number of cells in the range 0–10^6^ were suspended in 100 µL PBS, drawn up into a syringe and connected with a tube to the vessel. In each MPI data acquisition, the scanner ran continuously for 29 minutes, obtaining a new averaged full 3D dataset every 2.15 s. In each acquisition, the initial measurements were of the empty scanner only; these data were used for background correction. After two minutes the vessel was moved to the FOV centre, measuring only MNPs diluted in PBS without contact to cells. After one more minute the THP-1 cells were injected to the MNPs (time point defined as *t* = 0). For each time point the free (***c***_**1**_) and cell-bound (***c***_**2**_) MNP amounts were reconstructed. The reconstructed MPI signals were summed in the region of interest (ROI) around the nominal sample positions and converted into iron masses of the free (*m*_Fe,*free*_) and cell-bound (*m*_Fe,*cell*_) MNP distribution. The relative deviation of the total iron amount compared to the initially determined iron mass determined before cell-injection (*m*_Fe,0_) was calculated $\Delta {m}_{{\rm{Fe}}}=\frac{({m}_{{\rm{Fe}},free}+{m}_{{\rm{Fe}},cell})-{m}_{Fe,0}}{{m}_{{\rm{Fe}},0}}$ for each frame.

## Results

**Figure 1:**
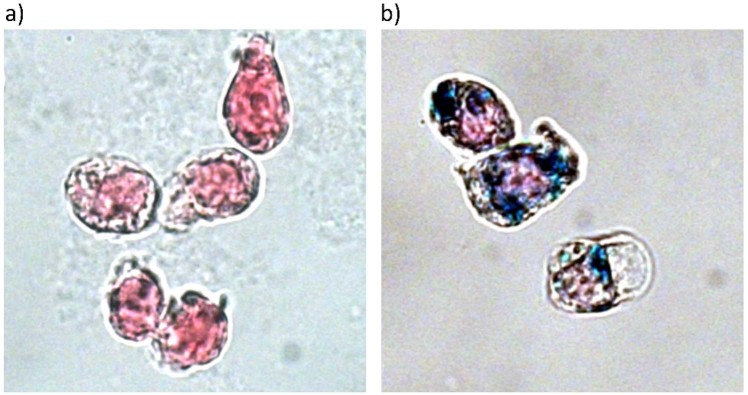
Light microscopy images of THP-1 cells after iron blue staining incubated without (**a**) and with (**b**) Synomag at an iron concentration of 0.5 mM for 15 min. Iron was visualized by Prussian Blue staining and nuclei with Nuclear Fast Red staining.

Images of light microscopy of unloaded THP-1 cells (a) and cells loaded for 15 min with 0.5 mM synomag (b) after Prussian blue staining and nuclear fast red staining are shown in Fig. 1. Light microscopy revealed that after this short incubation period a considerable amount of MNPs was present in the cytoplasm of THP-1 monocytes, indicating rapid uptake.

**Figure 2:**
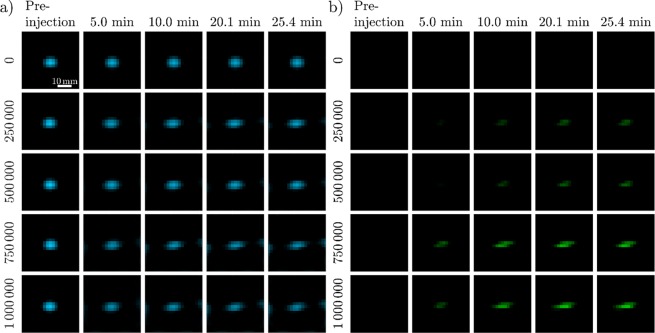
Reconstructed MPI images of the MNP distribution as a function of time of free (**a**) and cell-bound (**b**) MNPs in contact with THP-1 cells. Displayed are summed up intensities along the z-axis to visualize the 3D datasets. The same scaling was used for all images. The first column displays the reconstruction before injection of the cells (number of cells displayed on the left), in which only MNPs diluted in PBS were located in the FOV. The following columns show representative time frames after the injection with an increasing intensity of cell-bound MNPs at the same location compared to free MNPs.

The results of the *in-vitro* MPI experiment are displayed in Fig. 2. MPI reconstructions of free (a) and cell-bound (b) MNP-distributions of representative time frames are shown before and after injection of THP-1 cells. The sum of the intensities along the z-axis is presented in each case, to visualize the 3D dataset in a 2D image. The control measurement performed by injecting PBS without any THP-1 cells, showed no significant influence on the reconstructed images of free MNPs and no detection of cell-bound MNPs. Qualitatively, there were no significant differences detected in the reconstructions of free MNPs at different time steps. These were only visible when high numbers of cells were injected. Minor boundary artifacts can be observed starting at about 10 min after injection. Increasing the number of injected cells, led to a formation of cell-bound MNPs at the same position as the free MNPs with increasing intensities over time.

**Figure 3:**
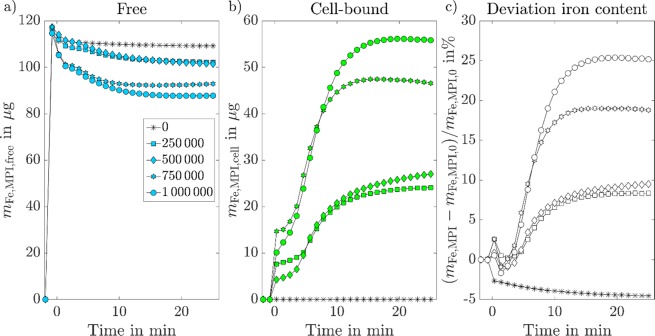
Quantified iron quantities of free (**a**) and cell-bound MNPs during initial MNP-THP-1 cell contact. With an increasing number of injected cells, a stronger increase of cell-bound MNPs and decrease of free MNPs was detected. (**c**) displays the relative deviation of the total iron amount (free+cell-bound MNPs) from the initially quantified amount of free MNPs before the injection. For better visualization, only a fraction of all available data points was incorporated in the figure.

The quantified iron amounts of free and cell-bound MNPs were extracted from the reconstructions and are displayed over time in Figs. 3a,b. After cell-injection, the amount of free MNPs decreases over time. The more cells were injected, the stronger the measured decrease of free MNPs. Simultaneously, the quantified values of cell-bound MNPs increases over time, also scaling with the number of injected cells. Saturation was reached after 10–15 minutes for all measurements. For each measurement, the quantified values of cell-bound MNPs were determined ≈30 minutes after injection and the average loading per cell was calculated to be *m*_Fe_ = (67 ± 19) pg.

The relative deviation of the total iron amount (Δ*m*_Fe_) is displayed over time in Fig. 3c. Overall, the deviation increases with the number of injected cells. The largest deviation was determined after injecting 10^6^ THP-1 cells, overestimating the total iron mass by 25.4%. The control experiment, performed by injection of PBS without cells, showed no significant changes in the quantified free and cell-bound iron masses. However, there was a small decrease below 5% of the free iron mass within the whole acquisition time. All reconstructed MPI images can be found as animated versions together with the quantification results in the Supplementary Data.

**Figure 4:**
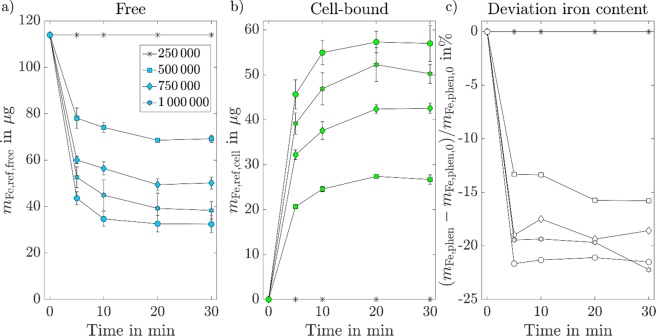
Quantified iron amounts acquired via the phenanthroline-based iron assay method for varying incubation times. Each data point represents the mean value from three independent measurements with the standard deviation visualized as error bars. Note that the lines connecting the dots do not represent measurement data and are only shown for better visualization. Due to the required washing steps during the phenanthroline-based iron assay method, some MNPs are lost, leading to an underestimation of the total iron mass by up to −22%, especially regarding the free MNPs.

The quantified iron amounts of the reference experiments, performed using the phenanthroline-based iron assay method, are displayed in Fig. 4. Each data point represents the averaged iron mass acquired from three independent measurements with the standard deviation visualized as error bars. Qualitatively, similar behaviour as in the MPI measurements can be observed. The free amounts of MNPs were decreasing over time until a saturation is reached after about 10–15 minutes. Simultaneously, the amount of cell-bound MNPs increased. The higher the number of injected cells, the stronger this effect was observed. After 30 minutes of MNP-cell exposure the average iron mass per cell was determined to be *m*_Fe_ = (80 ± 22) pg per cell. Figure 4c displays the relative deviation of the total iron mass, showing an underestimation of the total iron amount of up to −22%. These deviations are likely caused by the required washing steps (removal of the supernatant from the cell pellet and evaporation using a speedvac), which lead to losses of unbound MNPs and hence an underestimation of free MNPs.

## Discussion

The central finding of this study is that the distribution of free and cell-bound Synomag can be separated by our extended MPI image reconstruction based on different magnetic signals. This was achieved by incorporating the different magnetic signals generated by Synomag diluted in water and after cellular internalization into the MPI image reconstruction. This allowed for both the visualization and quantification of the cellular uptake of Synomag by THP-1 cells with a high temporal resolution of 2.15 s.

A decrease in the amount of reconstructed free MNPs and an increase in the amount of cell-bound MNPs over time supports the assumption that MNPs were accumulating in the cells. A similar pattern was observed when MPI results were qualitative compared with the results acquired with the reference experiment (phenanthroline-based iron assay). Both show a consistent decrease of free and increase of cell-bound MNPs until saturation is reached after about 10–15 minutes. This rapid cellular uptake of electrostatically stabilized MNPs is in accordance with previous studies using different particle systems^23^. There is a relative difference (23.8%) between the quantified values of cell-bound MNPs determined by MPI and the phenanthroline-based iron assay. Overall the MPI results acquired from measurements with high number of injected cells agree better with the phenanthroline results compared to low number of injected cells. This is probably due to interference from background noise or other signal sources, which, at very low concentrations of cell-bound MNP and the associated weak raw signal, makes exact identification and quantification difficult. Strong differences were observed when comparing the quantified values of free MNPs. These were likely the result of the loss of MNPs during the required washing and vacuum evaporation steps associated with the phenanthroline-based iron assay. As these steps mainly affect unbound MNPs, the amount of free MNPs was underestimated.

The deviation of the total iron amount determined by MPI increased over time and correlated with the number of injected cells. These deviations can only partly be attributed to technical issues, such as heating of hardware components and thermal drifts of the background signal, which could have caused the small deviations below 5% in the control experiment (as determined from injection of PBS without cells). The impact of thermal drifts could be minimized by an advanced background signal removal technique^34,35^. We assume the main reason for the deviation was caused by MNPs whose magnetic properties deviate from the states “free MNP” and “cell-bound MNPs”, which we used in this proof-of-principle study. The detection of signals generated by additional states leads to quantification errors and could also explain the imaging artifacts occurring at the edges of the FOV after 10 minutes in some of the reconstructed images^30,36^. We have previously shown, that MNPs undergo multiple changes of magnetic behaviour during cellular uptake^23,24^. At initial contact, MNPs are interacting with the pericellular matrix, a highly hydrated mesh consisting mainly of complex carbohydrates, such as glycosaminoglycans. This is followed by the initiation of a process known as endocytosis. Endocytosis is the process of internalizing extracellular substances which are then processed by a variety of intracellular vesicles such as endosomes and lysosomes. Later, MNPs are increasingly packed densely inside endosomes and finally degraded in the acidic environment of the endolysosomes^37^. All these stages seemed to be characterized by a distinct magnetic signature that can theoretically each be registered by a separate SF acquisition^23,24^. The magnetic signal changes between these states are smaller compared to the changes between the free and the cell-bound state used in this proof-of-principle study. The prerequisite for the detectability of such small differences and thus the differentiability of the individual states is the further improvement of the MPI sensitivity, which could be achieved by advanced hardware components and background correction methods^33,38^. Additionally, the creation of such a SF matrix would complicate the image reconstruction from a mathematical point of view but could be adapted to the respective experimental research question. Further studies are required to investigate the ability of differentiating these magnetic signal changes by multi-color MPI. This also includes studies investigating the influence of biological media and the formation of a protein corona around the MNP^39^. These processes affect the interactions between MNPs and cells, changing their uptake behaviour, and might also affect the magnetic signals of MNPs and hence the MPI performance^40–42^.

A different approach to include multiple states in the MPI image reconstruction would be to take the reconstructed signals gained from a two-state system as presented here. The respective MPI signals could then be correlated with a priori knowledge of the measured sample to generate a calibration curve of the cellular uptake process. A similar approach was presented for viscosity quantification using multi-contrast MPI^32^.

Previous studies have shown that by analysing the effects of MNPs on relaxation rates, MRI also enables the differentiation between free and cell-bound MNPs^43,44^. MRI differs from MPI by a higher spatial resolution and the simultaneous acquisition of the anatomy of the examined tissue, which is not imaged at MPI. However, MPI provides higher temporal resolutions of seconds or milliseconds compared to several minutes for MRI. Additionally, MPI detects signals generated by the MNPs directly, which enables a more reliable quantification, while MRI detects the effects of MNPs on the relaxation rates, which are difficult to distinguish from other sources (air, susceptibility changes, physiological or pathological changes, etc.) and require a reference scan before MNP injection for quantification^45^.

It was shown, that MPI can not only be used to image and quantify the spatial distribution of MNPs, but also to extract functional information about the environmental conditions such as the binding state based on the simple assumption of a two-state model. Overall imaging the cellular uptake process was possible for cell numbers down to 2.5 · 10^5^ with a temporal resolution of 2.15 s. This technique can be adapted for other cell types and MNP systems. This would permit for imaging of nanoscale processes, for example MNP internalization without damaging or destroying the cells. Improvements might be achieved by adapting the reconstruction process and by extending the two-state model as discussed above, which need to be investigated in more detail in future studies.

## Conclusion

This study demonstrates the potential of MPI to separate the signals of free and cell-bound MNPs, within a mixed sample. This technique can be used to image and quantify the uptake of MNPs into cells starting directly from initial MNP-cell contact thereby providing information about the dynamics of cellular uptake. It is assumed that the uptake dynamics of MNPs *in vivo* correlates with pathological changes of diseased tissue, in particular with the permeability of the vasculature and the infiltration of immune cells. Therefore, further development of this technique is anticipated, which might result in highly interesting opportunities for future diagnostic applications.

## Supplementary information

Supplementary Information.

Supplementary Information 2.

Supplementary Information 3.

Supplementary Information 4.

Supplementary Information 5.

Supplementary Information 6.
