# Supplementary figures and images for: Cellular uptake of magnetic nanoparticles imaged and quantified by magnetic particle imaging
Source: Sci Rep. 2020 Feb 5;10:1922. doi: 10.1038/s41598-020-58853-3 (PMC7002802; doi:10.1038/s41598-020-58853-3)

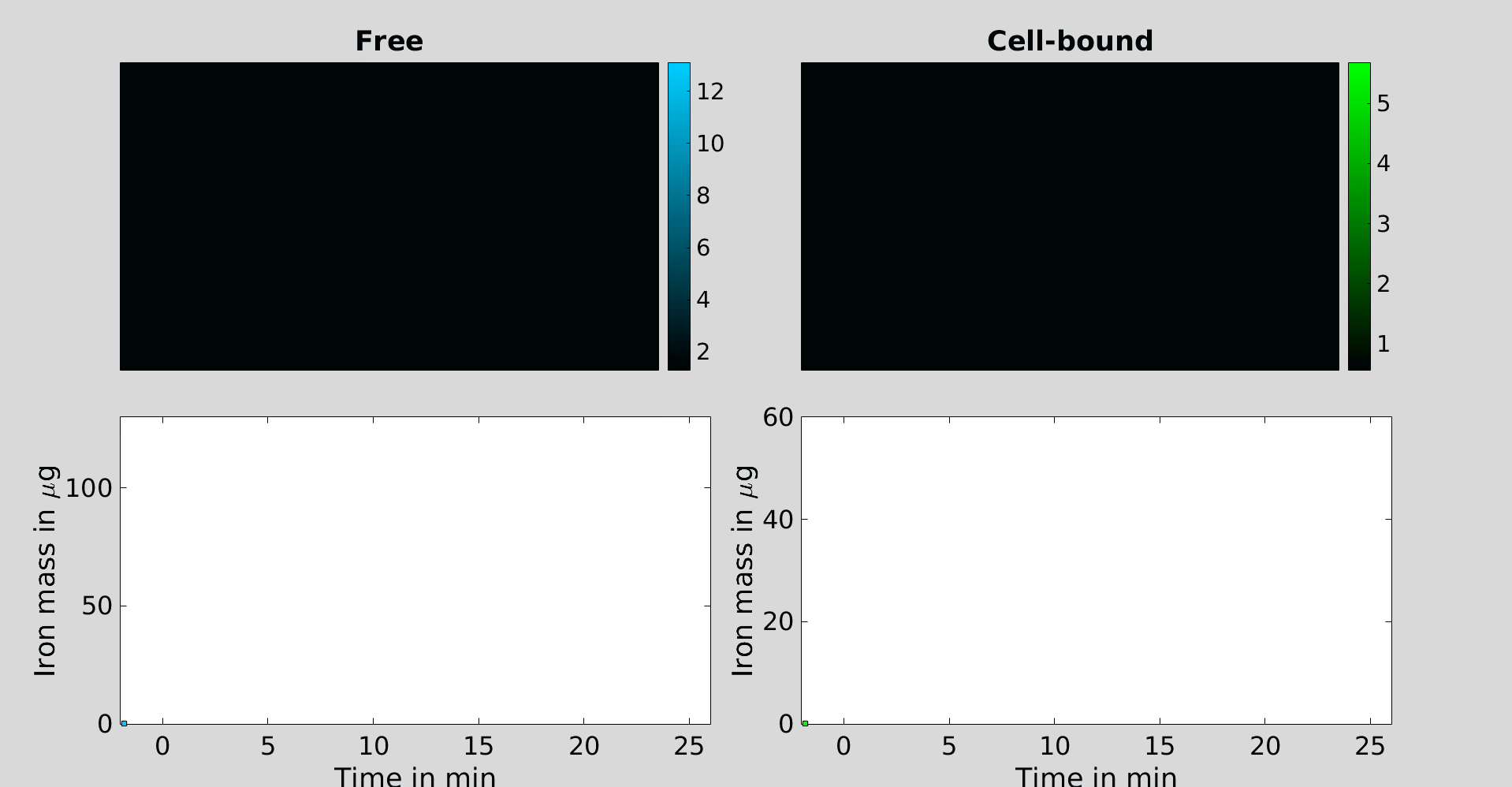

Supplement: Supplementary file 1 — Supplementary Information. [file 41598_2020_58853_MOESM1_ESM.gif]

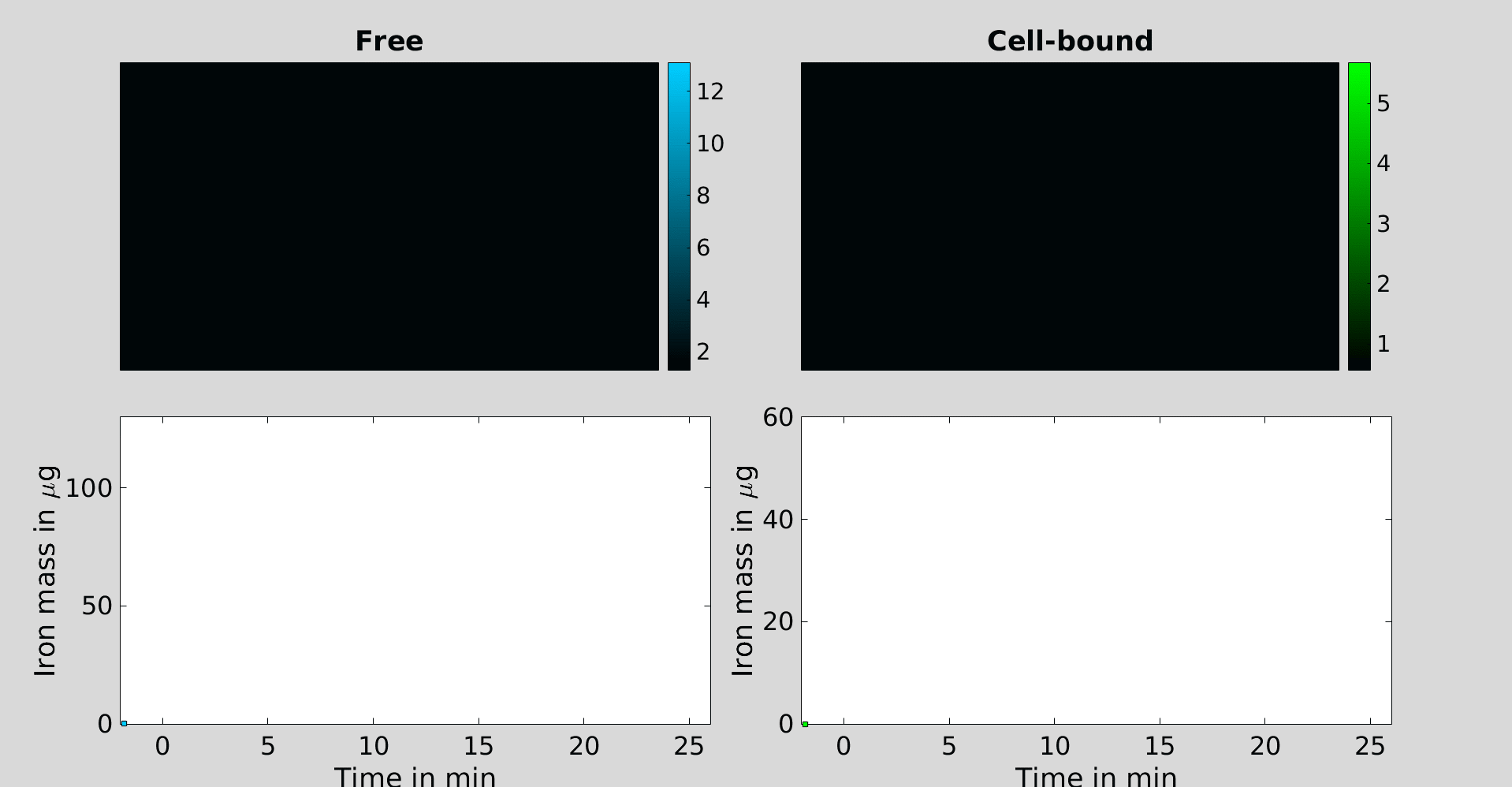

Supplement: Supplementary file 3 — Supplementary Information 3. [file 41598_2020_58853_MOESM3_ESM.gif]

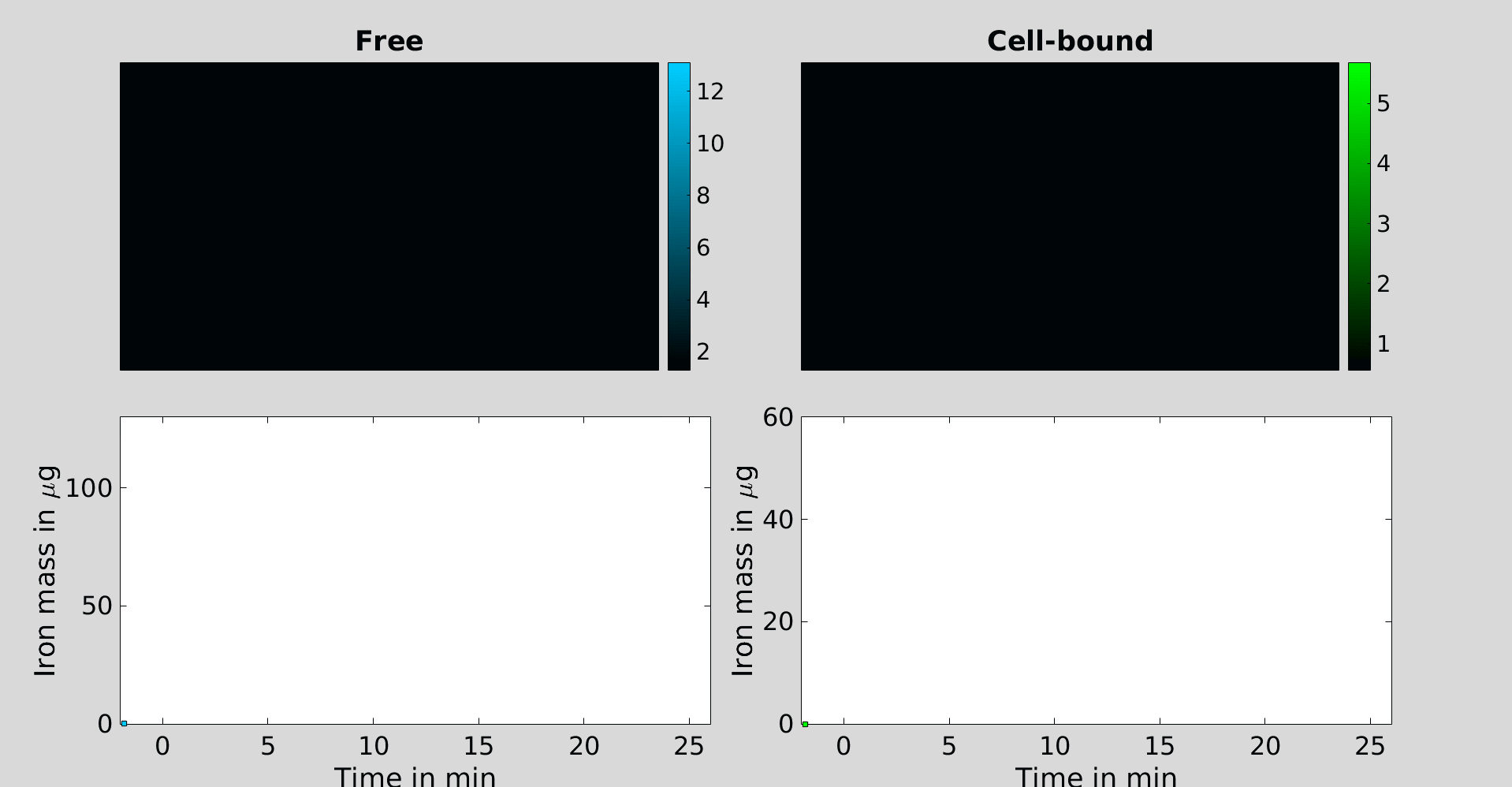

Supplement: Supplementary file 4 — Supplementary Information 4. [file 41598_2020_58853_MOESM4_ESM.gif]

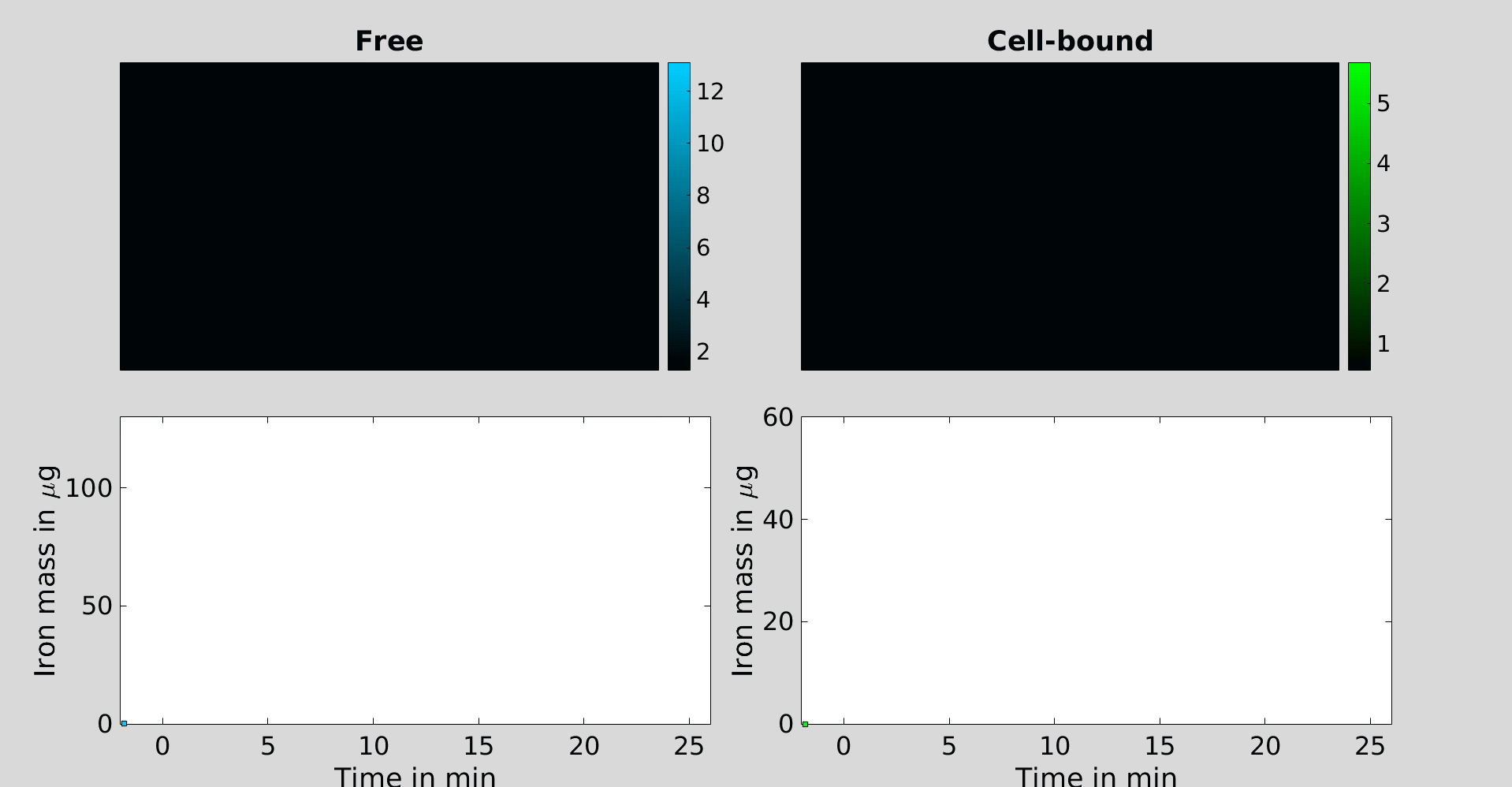

Supplement: Supplementary file 5 — Supplementary Information 5. [file 41598_2020_58853_MOESM5_ESM.gif]

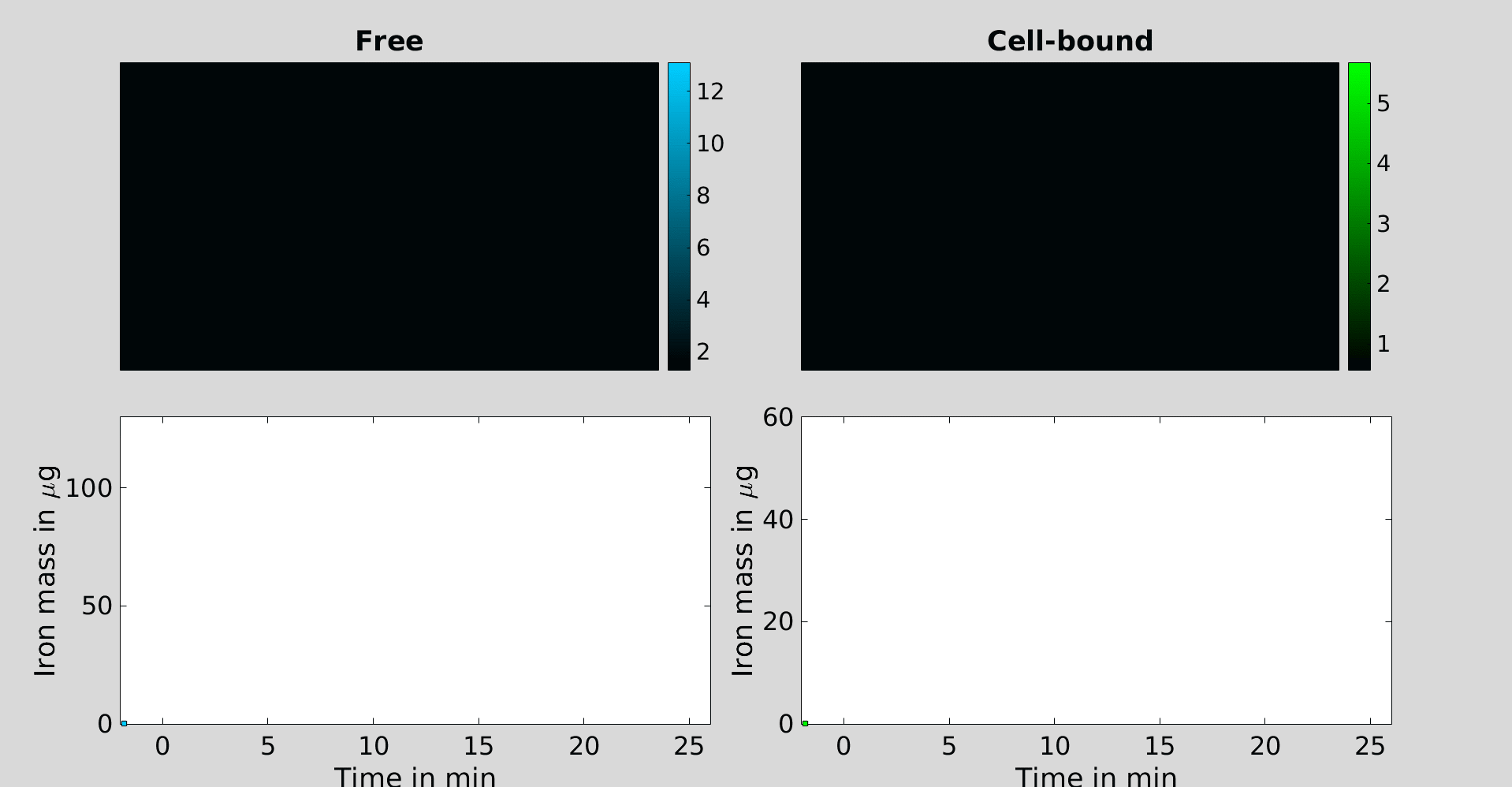

Supplement: Supplementary file 6 — Supplementary Information 6. [file 41598_2020_58853_MOESM6_ESM.gif]
