# Supplementary material for: Cellular uptake of magnetic nanoparticles imaged and quantified by magnetic particle imaging
Source: Sci Rep. 2020 Feb 5;10:1922. doi: 10.1038/s41598-020-58853-3 (PMC7002802; doi:10.1038/s41598-020-58853-3)
Supplement: Supplementary file 2 — Supplementary Information 2. [file 41598_2020_58853_MOESM2_ESM.pdf]

SUPPLEMENTARY METHODS

Hendrik Paysen[a*], Norbert Loewa[a], Anke Stach[b,c], James Wells[a], Olaf Kosch[a], Shailey Twamley[b,c], Marcus R. Makowski[b,d], Tobias Schaeffter[a], Antje Ludwig[b,c,e] and Frank Wiekhorst[a]

[a] *Physikalisch-Technische Bundesanstalt, Berlin, Germany*

[b] *Charité - Universitätsmedizin Berlin, corporate member of Freie Universität Berlin, Humboldt-Universität zu Berlin, Berlin Institute of Health, Medizinische Klinik für Kardiologie und Angiologie, Campus Mitte, Berlin, Germany*

[c] *DZHK (German Centre for Cardiovascular Research), partner site Berlin, Germany*

[d] *Technical University Munich, Germany*

[e] *Charité - Universitätsmedizin Berlin, corporate member of Freie Universität Berlin, Humboldt-Universität zu Berlin, Berlin Institute of Health, Klinik für Radiologie, Berlin, Germany*

[*] *Corresponding author, email: hendrik.paysen@ptb.de*

MPI measurement parameters

System function parameters	
**Parameter**	**Value/description**
**Drive fields**	12/12/12 mT
**Gradient strength**	0.6/0.6/1.2 T/m
**System function grid**	21x21x12
**FOV**	42x42x24 mm³
**Averages**	100
**Background correction**	Background increment 21; 5 BG repetitions
**Data acquisition software**	Paravision 6.0 (Bruker)
**Data processing**	Matlab 2015a (Mathworks)

MPI measurement parameters in-vitro study	
**Parameter**	**Value/description**
**Drive fields**	12/12/12 mT
**Gradient strength**	0.6/0.6/1.2 T/m
**Averages**	100 (2.15 s acquisition time)
**Repetitions**	800 (total acquisition time 29 min)
**Receive unit**	Gradiometric coil
**Background correction**	Subtraction of empty measurements
**Data acquisition software**	Paravision 6.0 (Bruker)
**Data processing**	Matlab 2015a (Mathworks)

Reconstruction parameters

A decisive role for MPI image reconstruction is the choice of the reconstruction parameters. In general, the MPI imaging equation is formulated as an underdetermined linear system. Therefore, the existence of a unique solution cannot be guaranteed. Hence, a least-squares problem is formulated to guarantee a solution, which is solved by the Kaczmarz algorithm with Tikhonov regularization. However, the number of frequency components used in the reconstruction, the number of iterations and the regularization parameter are variable and might have severe influence on the qualitative and quantitative values of the reconstructed images. In most MPI studies these parameters are chosen manually. Here, we performed a reconstruction parameter study in combination with a priori knowledge of the particle distribution to determine a full set of reconstruction parameters. The final reconstruction parameters are then used for the reconstructions of all measurement data to allow a quantitative comparison. In the following the procedure will be explained.

A time frame before injection of the cells was chosen for which the quantitative particle distribution is known. After background correction and truncation of the frequency components (>60 kHz), multiple image reconstructions were performed using the same

measurement data with varying reconstruction parameters. The number of frequency components (FCs) was varied between $25 - 2000$, sorted by the SNR of the SF, the relative regularization parameter λ (see [1]) was varied between $5 \cdot 10^{-5} - 5 \cdot 10^{0}$ and the number of iterations between $1 - 1000$. The intensities in a region of interest (ROI) around the nominal sample position were summed up and converted into the respective iron masses. Since the particle distribution is known, the difference of the reconstructed iron mass and the nominal iron mass can be calculated $\Delta m_{Fe} = m_{Fe,reco} - m_{Fe,nominal}$ for the free and cell-bound particle distributions respectively, which is visualized as a function of the reconstruction parameters in figure S1. The reconstruction parameters will be chosen to minimize Δm_{Fe} for both particle distributions. The final parameters are listed in the table below.

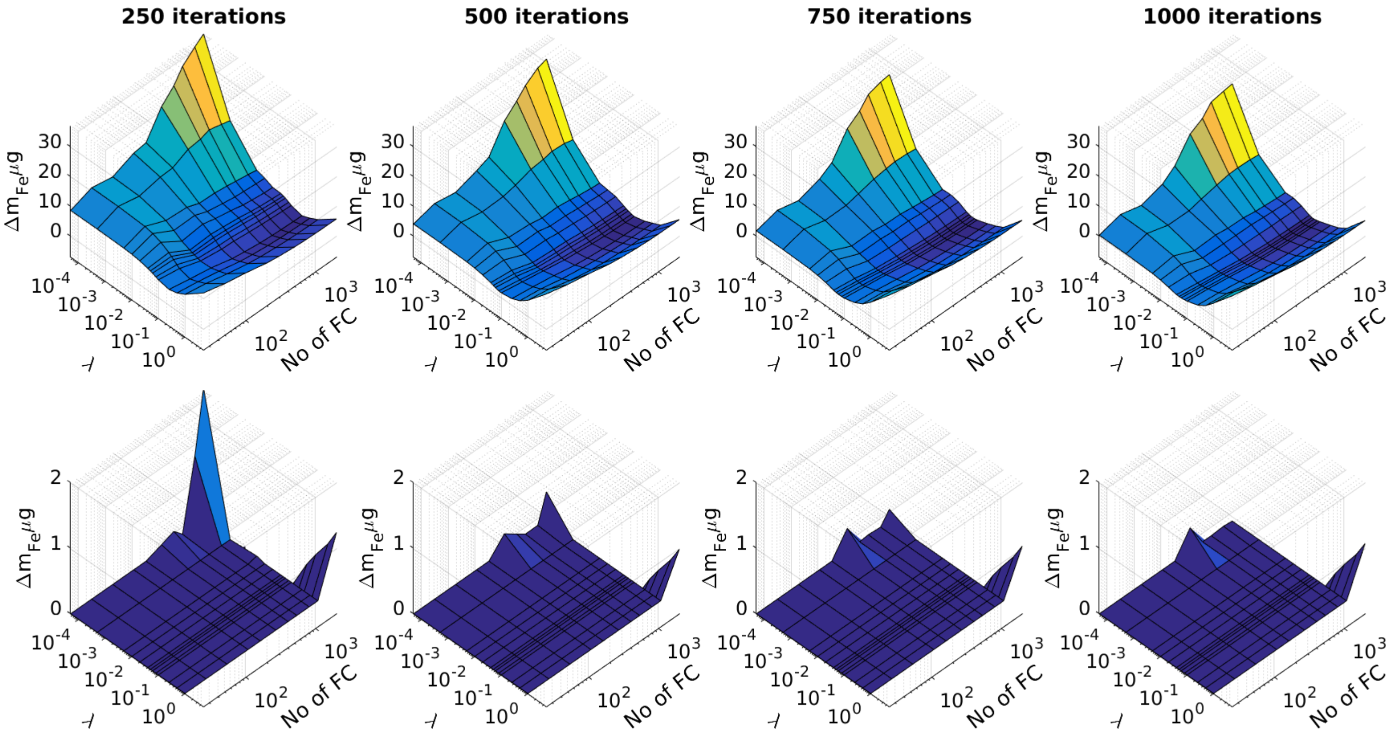

Figure S1: Visualization of reconstructed iron masses of free and cell-bound particle distributions determined in a ROI for the in-vitro study. Based on a priori knowledge of the nominal particle distributions, the difference to the reconstructed iron mass Δm_{Fe} was calculated and is minimized to determine the final reconstruction parameters used for all measurements.

**MPI reconstruction parameters in-vitro study**	
**Parameter**	**Value/description**
**Number of FC**	500
**Reconstruction algorithm**	Kaczmarz
**Regularization**	0.04
**Iterations**	1000

Supplementary references

[1] J. Weizenecker, J. Borgert, and B. Gleich, "A simulation study on the resolution and sensitivity of magnetic particle imaging.," *Phys. Med. Biol.*, vol. 52, no. 21, pp. 6363–6374, 2007.
